# Oral Frailty as a Risk Factor for Malnutrition and Sarcopenia in Patients on Hemodialysis: A Prospective Cohort Study

**DOI:** 10.3390/nu16203467

**Published:** 2024-10-13

**Authors:** Kota Miyasato, Yu Kobayashi, Kiyomi Ichijo, Ryo Yamaguchi, Hiroyuki Takashima, Takashi Maruyama, Masanori Abe

**Affiliations:** Division of Nephrology, Hypertension and Endocrinology, Department of Medicine, Nihon University School of Medicine, Tokyo 173-8610, Japan; kmiyasato123@gmail.com (K.M.); toki.88.um.45@gmail.com (Y.K.); ichijo.kiyomi@nihon-u.ac.jp (K.I.); yamaguchi.ryo@nihon-u.ac.jp (R.Y.); takashima.hiroyuki@nihon-u.ac.jp (H.T.); maruyama.takashi@nihon-u.ac.jp (T.M.)

**Keywords:** hemodialysis, malnutrition, oral frailty, physical frailty, sarcopenia

## Abstract

Background: Oral frailty is a concept that encompasses various aspects of impaired oral function in elderly people, leading to reduced food intake and less dietary diversity, which can result in sarcopenia and physical frailty. However, there have been no studies on the relationship between oral frailty and malnutrition, sarcopenia, and physical frailty in patients on hemodialysis (HD). Methods: This prospective observational cohort study assessed the oral status of patients on HD. The patients were divided into an oral frailty group and non-oral frailty group using the Oral Frailty Index-8. Malnutrition was assessed using the Geriatric Nutritional Risk Index (GNRI), the Nutritional Risk Index for Japanese Hemodialysis Patients (NRI-JH), and the Short-Form Mini-Nutritional Assessment (MNA-SF). Sarcopenia was assessed using the Asian Working Group for Sarcopenia 2019’s criteria. Physical frailty was assessed using the Japanese version of the Cardiovascular Health Study criteria. One year later, the changes in nutritional status, sarcopenia, and physical frailty risk categories were compared between the oral frailty and non-oral frailty groups. Results: The study enrolled 201 patients (non-oral frailty group, 123; oral frailty group, 78). After 1 year, the oral frailty group had a significantly higher proportion of patients with worsening nutrition status (GNRI, *p* = 0.0011; NRI-JH, *p* = 0.0019; MNA-SF, *p* < 0.001) and sarcopenia (*p* = 0.0024). There was no significant between-group difference in the proportion of patients in a worse risk category for physical frailty after 1 year. Conclusions: Oral frailty predicts future malnutrition and the progression of sarcopenia in HD patients. In particular, our results strongly suggested that oral frailty was a strong determinant of worsening malnutrition and sarcopenia in HD patients aged ≥65 years.

## 1. Introduction

The global elderly population is increasing, and the proportion of elderly people is particularly high in Japan. Elderly people are more likely to have chronic diseases, which often lead to a reduced quality of life (QOL). Elderly people are at a higher risk of developing sarcopenia and physical frailty, both of which are associated with adverse health outcomes [1,2].

Dialysis patients have chronic kidney disease, one of the most common chronic diseases. Dialysis patients are known to be susceptible to malnutrition, sarcopenia, and physical frailty due to a combination of multiple factors, including chronic inflammation, oxidative stress, the accumulation of uremic toxins, metabolic acidosis, and nutrient loss from dialysis fluid, all caused by prolonged renal failure and dialysis. Malnutrition, sarcopenia, and physical frailty are common among dialysis patients, and they are also linked to mortality [3,4,5]. Reduced QOL and increased mortality among dialysis patients are significant problems, and improving this situation is an important issue [6].

A nutritious diet is the foundation of good health. In particular, elderly people must eat a diverse range of foods in proper amounts to preserve muscle strength, allowing them to stay active. Generally, as people age, their ability to chew, move their tongue, and swallow declines, which can interfere with maintaining a nutritious diet. This condition is called “oral frailty” and has recently received attention in the field of geriatrics. Oral frailty was proposed as a concept to capture the various aspects of declining oral function in elderly people [7]. In elderly people, aging can cause tooth loss, deterioration of oral hygiene, and a decline in oral functions such as chewing and swallowing. In addition, older people often lose interest in their oral health and are unable to maintain their oral functions. When oral function declines, the ability to eat becomes impaired, which can lead to decreases in both physical and mental function. Indeed, the importance of oral frailty has already been reported, and a survey by Tanaka et al. of the general elderly population found that the occurrence of physical frailty and sarcopenia over a 2-year period increased 2.4- and 2.2-fold, respectively, in those with oral frailty [7].

In the same way that many elderly people have chronic diseases, dialysis patients have chronic kidney disease, one of the most common chronic diseases. Although the concept of ‘oral frailty’ is targeted at elderly people, we expected that it might also be applicable to patients on hemodialysis (HD). Although there have been some studies on oral frailty in the community-dwelling elderly [7,8,9], there have been no relevant studies on patients on HD.

The aim of this study was to elucidate the association of oral frailty with nutritional status, sarcopenia, and physical frailty in patients on HD, which could then provide valuable insights into oral care for these patients.

## 2. Materials and Methods

### 2.1. Study Design and Participants

The study had a multicenter, prospective, observational cohort design and enrolled patients who were receiving three 3–5 h HD sessions per week. The inclusion criteria were an age ≥ 20 years and the ability to stand. The following exclusion criteria were applied: current hospitalization or state of being bedridden; malignancy; a concurrent infectious disease; a history of limb amputation; and the implantation of a cardiac pacemaker (which would interfere with the automated measurement of skeletal muscle mass). Patients on HD were enrolled and divided into two groups according to the presence of oral frailty at baseline, and outcomes for nutritional status, sarcopenia, and physical frailty after 1 year of follow-up were compared between the groups. This clinical cohort study was performed in accordance with the principles of the Declaration of Helsinki and was approved by the institutional ethics committee of Nihon University Itabashi Hospital. It was also registered with the University Hospital Medical Information Network (UMIN000053222). All the patients provided written informed consent.

### 2.2. Definition of Oral Frailty

At baseline, the oral health status was measured in all the patients using the Oral Frailty Index-8 (OFI-8), which is a questionnaire designed to help identify adults who may be at risk of oral frailty [9]. The questions asked are listed in Supplementary Table S1. The OFI-8 consists of eight items and has a maximum score of 11. A higher score indicates worse oral frailty. A total score of 0–2 points indicates “low risk”, a total score of 3 points indicates “moderate risk”, and a total score of ≥4 points indicates “high risk”. The patients with a score of ≥4 points were allocated to an oral frailty group and those with a score of ≤3 points to a non-oral frailty group. 

### 2.3. Outcome Measure

#### 2.3.1. Nutritional Status

Nutritional risk was assessed using the Geriatric Nutritional Risk Index (GNRI), the Nutritional Risk Index for Japanese Hemodialysis Patients (NRI-JH), and the Short-Form Mini-Nutritional Assessment (MNA-SF).

GNRI can be measured easily and is often used to evaluate nutritional status in patients on HD [3]. It is calculated using the serum albumin level, dry weight (DW), and ideal body weight (IBW) as follows:

GNRI = [14.89 × serum albumin (g/dL)] + [41.7 × (DW/IBW)]

where ideal body weight (IBW) = height (m)^2^ × 22

According to the GNRI score, patients can be divided into four risk categories: no risk (≥98), low risk (≥92, <98), moderate risk (≥82, <92), and high risk (<82) (Supplementary Table S2).

NRI-JH is often used to evaluate nutritional status in patients on HD in Japan [10] and is calculated using body mass index (BMI) and serum albumin, total cholesterol, and creatinine levels (Supplementary Table S3). Based on the NRI-JH total score, patients are categorized as low risk (0–7 points), medium risk (8–10 points), or high risk (11–13 points).

MNA-SF is designed for routine nutritional screening in geriatric care [11] and includes six questions about BMI, any recent weight loss, any change in appetite, mobility, psychological stress, and neuropsychological problems. The questions are listed in Supplementary Table S4. According to the MNA-SF score, patients are classified as being at low risk (12–14 points), moderate risk (8–11 points), or high risk (0–7 points).

After 1 year, we classified the risk category for each indicator as “improvement or no change” or as “worsening”. The rate of worsening was compared between the oral frailty group and the non-oral frailty group.

#### 2.3.2. Sarcopenia

Sarcopenia was diagnosed using the criteria developed in 2019 by the Asian Working Group for Sarcopenia (AWGS 2019), which includes both “case finding” and “diagnosis”. Patients with chronic kidney disease in the algorithm for “acute to chronic healthcare or clinical research settings” proceeded to the second step, namely, “Diagnosis” (Supplementary Figure S1) [12]. All the patients were assessed for skeletal muscle mass, muscle strength, and physical performance. Skeletal muscle mass was measured after an HD session [13], and was evaluated with the Skeletal Muscle Index (SMI). The SMI was calculated from appendicular skeletal muscle mass (ASM) and height using the following formula:

SMI = ASM (kg)/height (m)^2^

The AWGS 2019 cutoff values for the diagnosis of low skeletal muscle mass in sarcopenia are SMI < 7.0 kg/m^2^ in men and SMI < 5.7 kg/m^2^ in women and were measured by multifrequency bioelectrical impedance analysis (InBody 270^®^ body analyzer, InBody, Tokyo, Japan). Muscle strength was measured by handgrip; the AWGS 2019 cutoff for low muscle strength is <28.0 kg in men and <18.0 kg in women for a diagnosis of sarcopenia. Physical performance was evaluated using the 6 m walk test. AWGS 2019 recommends using usual gait speed to define reduced physical performance, the cutoff for which is <1.0 m/s for the diagnosis of sarcopenia. AWGS 2019 defines patients with low muscle mass and low muscle strength and those with low muscle mass and low physical performance as having sarcopenia. Patients with low muscle mass, low muscle strength, and low physical performance are defined as having severe sarcopenia (Supplementary Table S5). In this study, a change in the risk category after 1 year was classified as “improvement or no change” or “worsening”. The rate of worsening was compared between the oral frailty group and the non-oral frailty group.

#### 2.3.3. Physical Frailty

In 2012, an international consensus group defined physical frailty [14]. In Japan, the revised Japanese version of the Cardiovascular Health Study (J-CHS) criteria [15] is often used to assess physical frailty. We used these criteria in this study. The revised J-CHS criteria evaluate five conditions: weight loss, muscle weakness, exhaustion, slowness, and low activity (Supplementary Table S6). The patients were divided into three risk categories: normal (0 points), prefrailty (1 or 2 points), and frailty (3–5 points). After 1 year, changes in the risk category were classified as “improvement or no change” or “worsening”. The rate of worsening was compared between the oral frailty group and the non-oral frailty group.

### 2.4. Data Collection

Demographic and clinical data, including age, sex, duration of dialysis, and comorbidities (e.g., diabetes mellitus, ischemic heart disease, cerebrovascular disease, and osteoporosis) as well as laboratory measurements were collected from the medical records. Clinical dry weight (DW) was used as the measure of body weight. Ischemic heart disease was defined as a history of angina pectoris, myocardial infarction, percutaneous coronary intervention, or coronary artery bypass grafting. Cerebrovascular disease was defined as a history of cerebral hemorrhage or cerebral infarction. Osteoporosis was defined as a history of fragility fracture, a young adult mean of ≤70%, or treatment with bisphosphonates. 

The serum urea nitrogen was measured using ultraviolet absorption spectrophotometry. The serum creatinine was measured using an enzymatic assay. The serum albumin was measured using the revised bromocresol purple assay. The serum sodium, potassium, and chloride were measured using an ion-selective electrode assay. The total cholesterol was measured using the cholesterol oxidase and peroxidase enzymatic assay. The total iron binding capacity was measured using the colorimetric method. The intact parathyroid hormone was measured using an electrochemiluminescence immunoassay. The hemoglobin was measured using the sodium lauryl sulfate-hemoglobin detection assay. The serum calcium was measured by an enzymatic assay using the alpha-amylase reaction. The serum phosphate was measured using an enzymatic reaction. The C-reactive protein levels were measured using the latex agglutination immunoassay. Blood samples were obtained in the HD group before initiation of the first weekly dialysis treatment. The single-pool Kt/V urea in one dialysis session was calculated [16].

### 2.5. Statistical Analysis 

Analyses were performed for all the patients, followed by subgroup analyses according to whether the age was ≥65 years or <65 years. The data are expressed as the mean ± standard deviation or as the median (interquartile range) as appropriate. Continuous variables were compared between the study groups using Student’s *t*-test or the Mann–Whitney *U* test and categorical variables using the chi-squared test or Fisher’s exact test. Furthermore, we investigated factors potentially associated with the prevalence of oral frailty. A univariate logistic regression analysis was performed using the OFI-8 score as the dependent variable and all the clinical and nutritional variables as independent variables. A multiple logistic regression analysis was then performed to identify the risk factors for oral frailty, using all the parameters that had *p* < 0.1 in the univariate analysis as independent variables. All the statistical analyses were performed using EZR version l.67 (Saitama Medical Center, Jichi Medical University, Saitama, Japan), which is a graphical user interface for R version 4.20 (The R Foundation for Statistical Computing, Vienna, Austria). More precisely, it is a modified version of R commander designed to add the statistical functions frequently used in biostatistics. A *p*-value of <0.05 was considered statistically significant [17].

## 3. Results

### 3.1. Prevalence of Oral Frailty in Patients on HD

**Figure 1:**
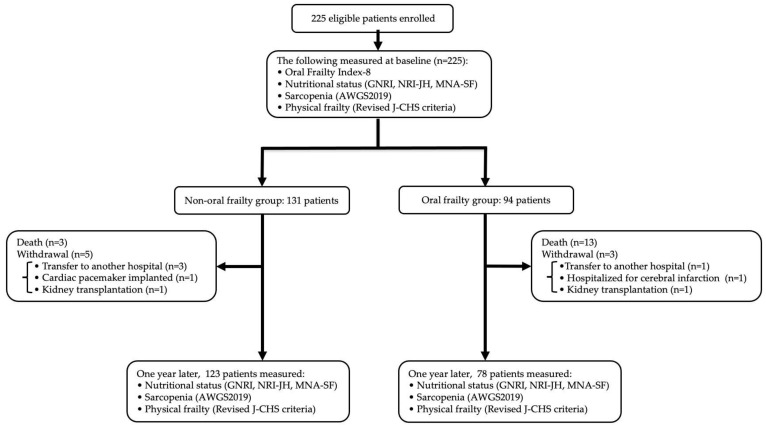
Flow chart for allocated patients into the non-oral frailty group and the oral frailty group. AWGS, Asian Working Group for Sarcopenia; GNRI, Geriatric Nutritional Risk Index; J-CHS, Japanese version of the Cardiovascular Health Study; MNA-SF, Short-Form Mini-Nutritional Assessment; NRI-JH, Nutritional Risk Index for Japanese Hemodialysis Patients.

**Figure 2:**
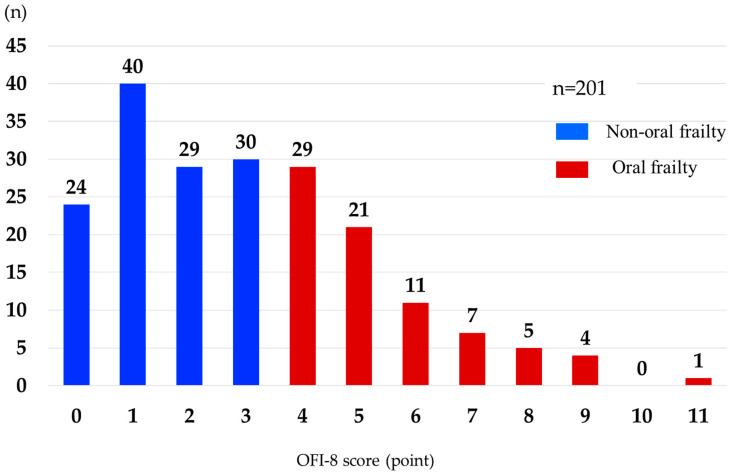
Distribution of OFI-8 scores. OFI-8, Oral Frailty Index-8.

An overview of the process used to select the study participants is shown in Figure 1. In total, 225 eligible patients were enrolled. Eight patients were excluded because of their transfer to another hospital (*n* = 4), the cessation of HD owing to kidney transplantation (*n* = 2), hospitalization for cerebral infarction (*n* = 1), or cardiac pacemaker implantation (*n* = 1). Sixteen (7.4%) of the remaining 217 patients died during the one-year follow-up period, leaving the data for 201 patients for analysis. Baseline OFI-8 and changes in each index were evaluated in these 201 patients (139 men, 62 women; mean age, 69.8 ± 13.2 years). The distribution of the OFI-8 scores is shown in Figure 2. Oral frailty was identified in 78 patients, giving a prevalence rate of 38.8%.

### 3.2. Characteristics of Patients in the Oral Frailty and Non-Oral Frailty Groups at Baseline

Patients in the oral frailty group were significantly more likely to be older and to have lower creatinine levels and diagnoses of osteoporosis. There was no significant difference in the sex distribution, duration of dialysis, dialysis time, BMI, blood pressure, heart rate, serum urea nitrogen, hemoglobin, albumin, corrected calcium, phosphate, total iron binding capacity, intact parathyroid hormone, total cholesterol, C-reactive protein, Kt/V, cause of end-stage kidney disease, or comorbidities, such as diabetes mellitus, ischemic heart disease, or cerebrovascular disease (Table 1).

Table 2 shows the distribution of each frailty category by GNRI, NRI-JH, MNA-SF, AWGS, and revised J-CHS criteria in the two groups. There was no significant difference in the GNRI or NRI-JH category between the two groups. The proportion at risk of malnutrition by the MNA-SF score was significantly higher in the oral frailty group than in the non-oral frailty group. The proportion with sarcopenia or severe sarcopenia was higher in the oral frailty group, as was the proportion with pre-frailty or frailty.

To identify factors associated with the severity of oral frailty, univariate linear regression analysis was performed using the OFI-8 score as the dependent variable and all clinical and nutritional variables as independent variables in all the patients (Supplementary Table S7). The OFI-8 score was significantly associated with older age, the presence of osteoporosis, lower serum creatinine, albumin, and total cholesterol levels, lower GNRI and MNA-SF scores, higher NRI-JH and revised J-CHS scores, and the severity of sarcopenia. A multivariate linear regression analysis was performed to identify the independent predictors of the OFI-8 score (Supplementary Table S7). Older age and revised J-CHS scores were independent predictors of the OFI-8 score in the adjusted model.

### 3.3. Changes in Risk Categories for Nutritional Status, Sarcopenia, and Physical Frailty in the Two Groups

**Figure 3:**
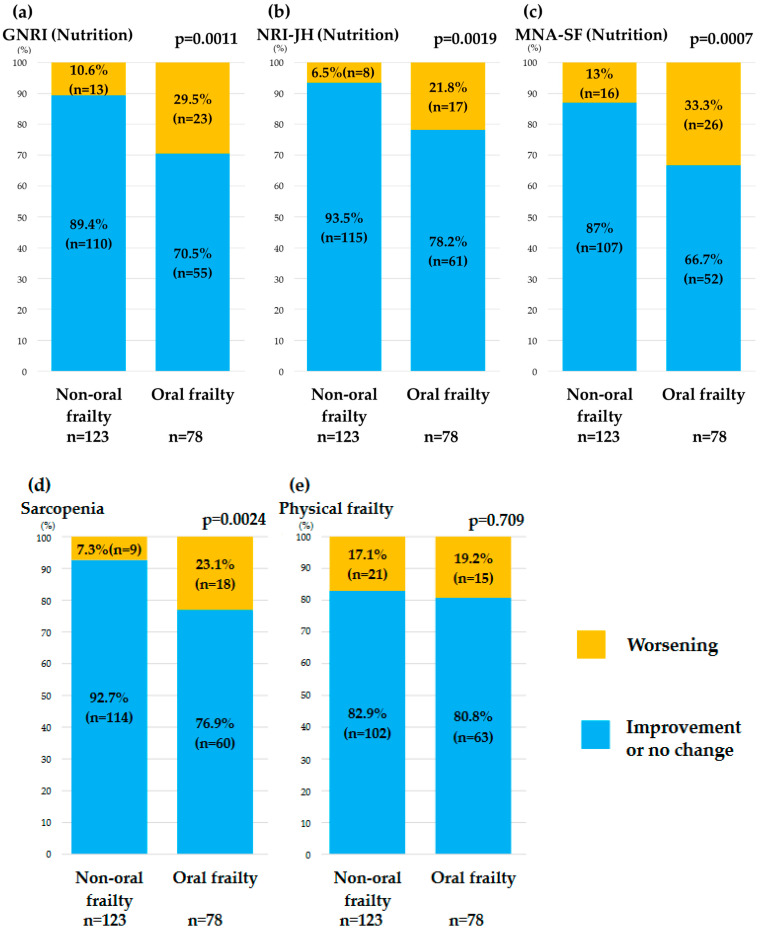
Comparison of changes in each indicator, including nutritional status (**a**–**c**), sarcopenia (**d**), and physical frailty (**e**) between the non-oral frailty group (OFI-8 ≤ 3) and the oral frailty group (OFI-8 ≥ 4). GNRI, Geriatric Nutritional Risk Index; MNA-SF, Short-Form Mini-Nutritional Assessment; OFI-8, Oral Frailty Index-8; NRI-JH, Nutritional Risk Index for Japanese Hemodialysis Patients.

Worsening in the GNRI, NRI-JH, and MNA-SF categories over 1 year was significantly greater in the oral frailty group than in the non-oral frailty group. After 1 year, worsening of the sarcopenia category was significantly greater in the oral frailty group. However, there was no between-group difference in the deterioration of physical frailty (Figure 3). 

### 3.4. Subgroup Analysis by Age

Subgroup analyses were performed according to whether the age was ≥65 years (elderly patients, *n* = 135) or <65 years (younger patients, *n* = 66). 

**Figure 4:**
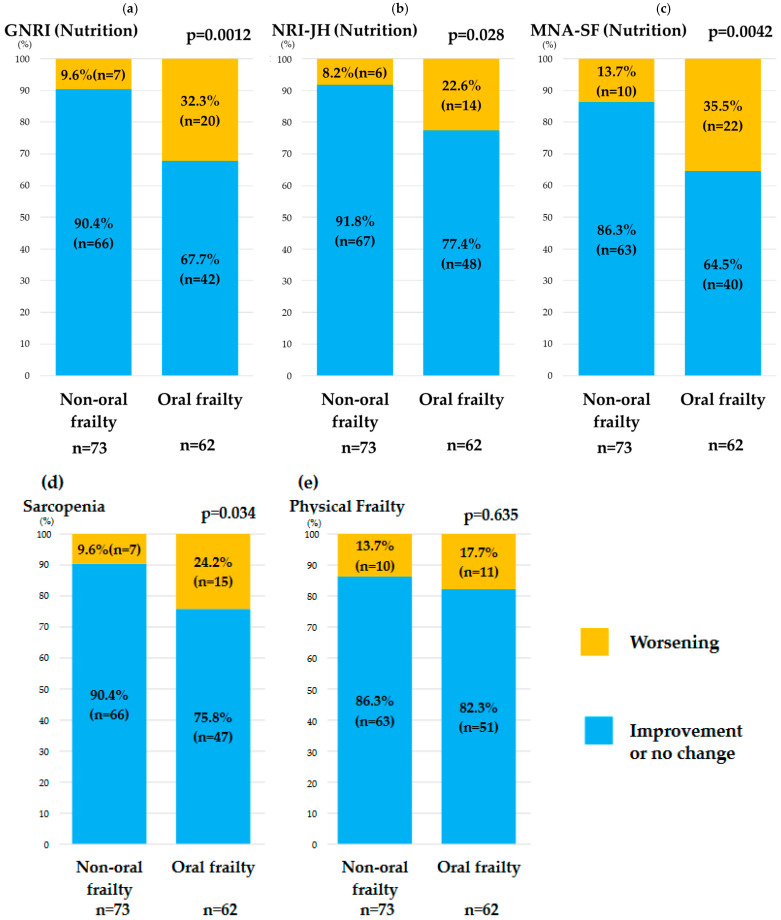
Comparison of changes in each indicator, including nutritional status (**a**–**c**), sarcopenia (**d**), and physical frailty (**e**), between the non-oral frailty group (OFI-8 ≤ 3) and the oral frailty group (OFI-8 ≥ 4) in patients aged ≥65 years. GNRI, Geriatric Nutritional Risk Index; MNA-SF, Short-Form Mini-Nutritional Assessment; OFI-8, Oral Frailty Index-8; NRI-JH, Nutritional Risk Index for Japanese Hemodialysis Patients.

In elderly patients, there was no significant between-group difference in baseline characteristics except for the proportion with physical frailty (Table 3 and Table 4). Worsening in the GNRI, NRI-JH, and MNA-SF categories over 1 year was significantly greater in the oral frailty group than in the non-oral frailty group. After 1 year, worsening of the sarcopenia category was significantly greater in the oral frailty group. However, there was no between-group difference in the deterioration of physical frailty (Figure 4).

**Figure 5:**
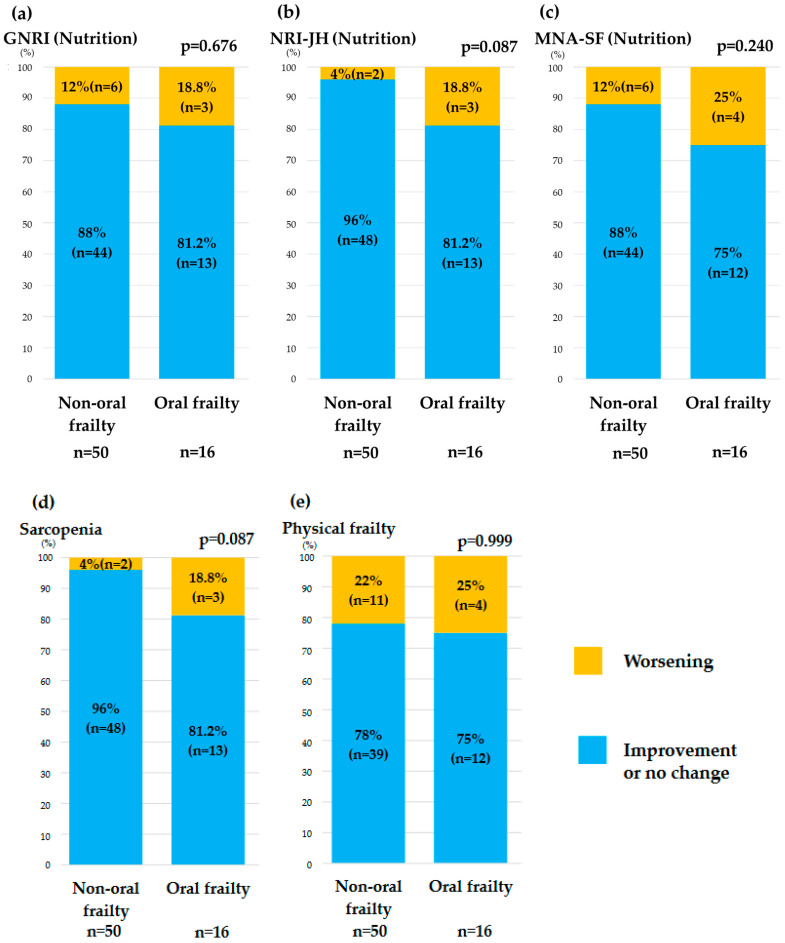
Comparison of changes in each indicator, including nutritional status (**a**–**c**), sarcopenia (**d**), and physical frailty (**e**), between the non-oral frailty group (OFI-8 ≤ 3) and the oral frailty group (OFI-8 ≥ 4) in patients aged < 65 years. GNRI, Geriatric Nutritional Risk Index; MNA-SF, Short-Form Mini-Nutritional Assessment; OFI-8, Oral Frailty Index-8; NRI-JH, Nutritional Risk Index for Japanese Hemodialysis Patients.

In the younger patients, those in the oral frailty group were significantly more likely to be older (Table 5). Younger patients in the oral frailty group had malnutrition as measured by GNRI and worse sarcopenia and physical frailty (Table 6). However, there was no deterioration in nutritional status, sarcopenia, or physical frailty 1 year later between the two groups (Figure 5).

## 4. Discussion

This study found that oral frailty in patients on HD was associated with aging, lower serum creatinine level, and comorbid osteoporosis. In turn, oral frailty was associated with the outcomes of malnutrition, sarcopenia, and physical frailty. Our patients on HD with oral frailty showed worsening nutritional status after 1 year of follow-up, as measured using GNRI, NRI-JH, and MNA-SF scores. However, although the sarcopenia risk category in patients with oral frailty was observed to worsen according to the AWGS 2019 criteria, there was no change in physical frailty according to the revised J-CHS criteria. These findings were seen in elderly patients but not in younger patients. Subgroup analysis did not reveal any significant between-group differences in baseline characteristics among elderly patients, except for physical frailty. However, oral frailty was identified to be a strong determinant of worsening malnutrition and sarcopenia in patients aged ≥65 years. This is the first study to show the associations of oral frailty with poor nutritional status and the increased risk of sarcopenia in patients on HD.

Oral frailty often leads to difficulties in chewing and swallowing, reduced food intake, and less dietary diversity, including lower protein intake. Motokawa et al. found that the intake of nutrients, including energy and fat, was significantly lower in the group with poor chewing ability than in the group with good chewing ability, as was the intake of many other food groups [18]. Two studies have investigated the relationship between oral frailty and dietary diversity [19,20]. Reducing food intake and dietary diversity can exacerbate malnutrition. In populations prone to sarcopenia, which includes patients on HD [4,21], malnutrition can further exacerbate sarcopenia, and the weakening of the muscles around the mouth can exacerbate oral frailty further. This vicious cycle worsens oral frailty in patients on HD. 

OFI-8 was designed for screening purposes and not as a diagnostic criterion. Oral frailty is reported to be associated with declines in mental and physical function, suggesting that it should be identified using a multidimensional approach [22,23]. For example, a study by Nagatani et al. identified oral frailty using six components, namely, the number of remaining teeth, chewing ability, tongue pressure, oral motor skills, and subjective difficulties in eating and swallowing, but did not include assessments of oral health-related behaviors and social participation [8]. In contrast, the OFI-8 includes key indicators of oral frailty, including denture use and chewing ability, as well as assessments of oral health-related behaviors and social participation. Although the OFI-8 lacks objective indicators, it is useful in terms of its convenience and comprehensiveness and is often used to detect oral frailty [9,24,25]. Although a detailed diagnosis of oral frailty includes dental evaluation, most dialysis facilities in Japan do not have in house dental departments or relationships with registered dental clinics [26]. Therefore, it is difficult to make a detailed diagnosis of oral frailty in all dialysis facilities. We believe that the OFI-8 is useful for identifying patients who require prevention or treatment for oral frailty. Therefore, we used it in our study. 

We intentionally used multiple nutritional indicators in this study. Many indicators can be used to detect patients with malnutrition. Some indicators include many survey items that are accurate but are too complicated for use in general dialysis facilities. Therefore, for the purposes of this study, we used GNRI, NRI-JH and MNA-SF, all of which are valid and relatively easy to measure. GNRI is often used to evaluate nutritional status in patients on HD and can predict mortality in these patients [3,27]. NRI-JH is a nutritional index that detects malnourished patients at high risk of death in patients on HD [10]. MNA-SF is recommended as a screening tool for nutritional status in HD patients and has been associated with 2-year mortality [28].

Patients on HD differ from those not on dialysis in that they are more susceptible to osteoporosis and periodontal disease. Osteoporosis is common in patients with chronic kidney disease, including those on HD, and is caused by various mechanisms, including chronic kidney disease/mineral and bone disorder and uremia. Fragile jawbones lead to reduced chewing ability. Periodontal disease is known to be more common and more severe in patients on dialysis than in healthy individuals [29] and leads to tooth loss, which is a risk factor for oral frailty. It has been reported that the accurate diagnosis and appropriate treatment of periodontal disease not only reduces the risk of oral infection and inflammation but also significantly extends survival in patients on HD [30]. Patients on HD are more susceptible to osteoporosis and periodontal disease and may be more prone to developing oral frailty compared to individuals not on HD.

There are a variety of methods that could potentially be used for the prevention and treatment of oral frailty outside of dental clinics. Mouth-opening exercises consisting of repetitions of fully opening the jaw and resting are an effective treatment for dysphagia [31]. Lingual exercise is reported to increase tongue muscle strength and improve swallowing pressure, airway protection, and tongue volume in stroke patients with acute or chronic dysphagia [32]. The CAMCAM program, in which participants listen to a lecture on oral health, nutrition, and diet while eating a lunch containing foods that strengthen chewing power, led to significant improvements in oral frailty and its severity [25]. Another program that consisted of instruction in oral hygiene, facial and tongue muscle exercises, and salivary gland massage significantly improved salivation and swallowing in older people [33]. By changing attitudes towards oral health, these methods can not only help prevent oral frailty, but also prompt visits to the dental clinic. Early dental treatment can prevent the serious consequences of dental neglect, including tooth loss, and contribute to the prevention of oral frailty. Our study paves the way for future interventions by healthcare providers in the prevention and treatment of oral frailty.

This study has several limitations. First, although we measured skeletal muscle mass as part of the assessment of sarcopenia, owing to the limitations of the equipment, which requires patients to hold a standing position for measurement of skeletal muscle mass, only those who could hold a standing position were included. The exclusion of patients who could not maintain a standing position for muscle mass measurements may have introduced selection bias. If these patients had been included, they could have provided valuable insights into more severe cases of sarcopenia. In addition, although the worsening of sarcopenia was found after 1 year of follow-up, the severity of physical frailty did not change in the oral frailty group. The 1-year follow-up period may not have been sufficient to observe significant changes in physical frailty. Therefore, further long-term studies are required. Second, the OFI-8 used to assess oral frailty in this study is not an objective measure, but a subjective self-report by patients. Therefore, it may not accurately reflect their true oral health status. To accurately assess oral health, it is necessary to take the time to conduct a detailed evaluation of dental condition, masticatory function, and swallowing function for each patient. Third, NRI-JH includes total cholesterol as a nutritional indicator, which may not accurately reflect nutritional status because patients taking statins may have lower total cholesterol levels. Therefore, we considered that NRI-JH alone could not be used as a reliable indicator for assessing nutritional status. For this reason, to comprehensively assess nutritional status, we also used GNRI and MNA-SF, which are not influenced by serum cholesterol levels and are unaffected in patients taking statins. Fourth, we could not compare mortality between the two groups. All-cause mortality is the most important hard endpoint and is an outcome that should be addressed. HD patients are known to have a higher incidence of sudden death compared with the general population [34]. The causes of sudden death in HD patients are diverse; although cardiac disease is the most common cause, other causes include arrhythmias due to hyperkalemia. Future studies are needed to confirm the association between oral frailty and mortality. Fifth, this study was conducted as an exploratory study because there had been no similar studies conducted in the past, making it difficult to estimate the sample size. Finally, because this study included only Japanese patients on HD, further investigation is needed to determine whether the results of this study are applicable in other populations with different dietary cultures.

## 5. Conclusions

Our findings show that oral frailty strongly predicts the worsening of malnutrition and sarcopenia in patients on HD. In particular, it was strongly suggested that oral frailty is a strong determinant of worsening malnutrition and sarcopenia in patients aged ≥65 years. Early assessment and the improvement of oral frailty might prevent the onset or worsening of nutritional status and sarcopenia and improve the prognosis of these patients.

## Figures and Tables

**Table 1:** Patient characteristics at baseline for all patients and for the oral frailty and non-oral frailty groups.

Variable	All Patients	Non-Oral Frailty Group	Oral Frailty Group	*p*-Value
Patients, *n* (male, %)	201 (69.2)	123 (69.1)	78 (69.2)	0.998
Age, years	69.8 ± 13.2	66.9 ± 13.7	74.4 ± 11.2	<0.0001
Duration of dialysis, months	60 (26–122)	57 (25–130)	67 (31–118)	0.576
Dialysis time, min	233 ± 23	235 ± 23	229 ± 24	0.090
Body mass index, kg/m^2^	22.5 ± 4.2	23.0 ± 4.3	21.7 ± 3.9	0.057
Systolic blood pressure, mmHg	158 ± 24	157 ± 25	161 ± 22	0.284
Diastolic blood pressure, mmHg	81 ± 14	82 ± 15	80 ± 14	0.459
Heart rate, bpm	73 ± 12	74 ± 11	72 ± 12	0.276
Serum urea nitrogen, mg/dL	60.1 ± 14.5	60.9 ± 14.3	58.9 ± 14.8	0.335
Creatinine, mg/dL	9.6 ± 2.8	10.0 ± 3.0	9.0 ± 2.5	0.025
Hemoglobin, g/dL	11.0 ± 1.2	11.1 ± 1.2	10.9 ± 1.2	0.214
Albumin, g/dL	3.5 ± 0.4	3.6 ± 0.3	3.5 ± 0.4	0.056
Corrected calcium, mg/dL	9.1 ± 0.7	9.0 ± 0.7	9.1 ± 0.7	0.896
Phosphate, mg/dL	5.1 ± 1.3	5.0 ± 1.3	5.1 ± 1.2	0.968
Intact PTH, pg/mL	138 (94–203)	138 (101–213)	137 (84–193)	0.408
C-reactive protein, mg/dL	0.16 (0.07–0.43)	0.16 (0.08–0.49)	0.18 (0.06–0.40)	0.929
Total iron binding capacity, μg/dL	265 ± 59	268 ± 59	260 ± 58	0.352
Total cholesterol, mg/dL	160 ± 33	164 ± 35	156 ± 30	0.114
spKt/V	1.42 ± 0.24	1.42 ± 0.24	1.41 ± 0.24	0.749
Cause of ESKD, *n* (%)				0.835
Hypertension	66	41 (20.4)	25 (12.4)	
Diabetic nephropathy	104	62 (30.8)	42 (20.9)	
Chronic glomerular nephritis	14	8 (4.0)	6 (3.0)	
Others	17	12 (6.0)	5 (2.5)	
Comorbidities, *n* (%)				
Diabetes mellitus	106 (52.7)	63 (51.2)	43 (55.1)	0.664
Ischemic heart disease	67 (33.3	43(35.0)	24 (30.8)	0.645
Cerebrovascular disease	85 (42.3)	46 (37.3)	39 (50.0)	0.082
Osteoporosis	91 (45.3)	45 (36.6)	46 (59.0)	0.002

ESKD, end-stage kidney disease; PTH, parathyroid hormone.

**Table 2:** Values for each indicator (nutritional status, sarcopenia, and physical frailty) at baseline for all patients and for oral frailty and non-oral frailty groups.

Indicator	All Patients	Non-Oral Frailty Group	Oral Frailty Group	*p*-Value
GNRI, %				0.145
No risk	41.3	47.2	32.1	
Low risk	23.4	22.8	24.4	
Moderate risk	26.9	23.6	32.1	
High risk	8.4	6.4	11.5	
NRI-JH, %				0.372
Low risk	88.6	91.1	84.6	
Medium risk	7.5	5.7	10.3	
High risk	3.9	3.2	5.1	
MNA-SF, %				0.048
Normal	66.7	73.2	56.4	
Risk of malnutrition	32.3	26.0	42.3	
Malnutrition	1.0	0.8	1.3	
Sarcopenia, %				0.0004
Normal	53.2	61.8	39.7	
Sarcopenia	23.9	24.4	23.1	
Severe sarcopenia	22.9	13.8	37.2	
Revised J-CHS, %				<0.0001
Normal	23.4	30.1	12.8	
Prefrailty	51.7	57.7	42.3	
Frailty	24.9	12.2	44.9	

GNRI, Geriatric Nutritional Risk Index; J-CHS, Japanese version of the Cardiovascular Health Study; MNA-SF, short-form mini-nutritional assessment; NRI-JH, Nutritional Risk Index for Japanese Hemodialysis Patients.

**Table 3:** Patient characteristics at the baseline in patients aged ≥65 years in the oral frailty and non-oral frailty groups.

Variable	All Patients	Non-Oral Frailty Group	Oral Frailty Group	*p*-Value
Patients, *n* (male, %)	135 (63.7)	73 (64.4)	62 (62.9)	0.860
Age, years	77.4 ± 7.5	76.3 ± 7.2	78.6 ± 7.7	0.071
Duration of dialysis, months	56 (24–114)	52 (19–83)	61 (31–116)	0.230
Dialysis time, min	227 ± 23	229 ± 23	225 ± 22	0.386
Body mass index, kg/m^2^	21.8 ± 3.8	21.9 ± 4.0	21.6 ± 3.7	0.721
Systolic blood pressure, mmHg	156 ± 24	153 ± 24	159 ± 23	0.104
Diastolic blood pressure, mmHg	77 ± 13	76 ± 14	78 ± 13	0.275
Heart rate, bpm	71 ± 12	71 ± 12	71 ± 11	0.953
Serum urea nitrogen, mg/dL	60.0 ± 14.6	61.6 ± 15.6	58.1 ± 13.3	0.164
Creatinine, mg/dL	8.8 ± 2.5	8.9 ± 2.7	8.8 ± 2.3	0.839
Hemoglobin, g/dL	11.1 ± 1.2	11.2 ± 1.2	10.9 ± 1.1	0.101
Albumin, g/dL	3.5 ± 0.4	3.5 ± 0.4	3.4 ± 0.4	0.323
Corrected calcium, mg/dL	9.1 ± 0.7	9.0 ± 0.6	9.1 ± 0.8	0.571
Phosphate, mg/dL	5.0 ± 1.3	5.0 ± 1.2	5.0 ± 1.3	0.538
Intact PTH, pg/mL	131 (83–200)	134 (87–216)	122 (83–197)	0.393
C-reactive protein, mg/dL	0.18 (0.08–0.47)	0.16 (0.09–0.52)	0.19 (0.07–0.41)	0.972
Total iron binding capacity, μg/dL	253 ± 50	250 ± 46	256 ± 54	0.499
Total cholesterol, mg/dL	159 ± 31	160 ± 32	156 ± 29	0.541
spKt/V	1.42 ± 0.26	1.42 ± 0.27	1.41 ± 0.26	0.769
Cause of ESKD, *n* (%)				0.277
Hypertension	48 (35.6)	26 (19.3)	22 (16.3)	
Diabetic nephropathy	70 (51.8)	39 (28.9)	31 (23.0)	
Chronic glomerular nephritis	6 (4.4)	1 (0.7)	5 (3.7)	
Others	11 (8.1)	7 (5.2)	4 (3.0)	
Comorbidities, *n* (%)				
Diabetes mellitus	72 (53.3)	40 (54.8)	32 (51.6)	0.732
Ischemic heart disease	49 (36.3)	30 (41.1)	19 (30.6)	0.281
Cerebrovascular disease	64 (47.4)	30 (41.1)	34 (54.8)	0.123
Osteoporosis	81 (60.0)	39 (53.4)	42 (67.7)	0.113

ESKD, end-stage kidney disease; PTH, parathyroid hormone.

**Table 4:** Risk categories for nutritional status, sarcopenia, and physical frailty at baseline in all patients aged ≥65 years and in the oral frailty and non-oral frailty groups.

Indicator	All Patients	Non-Oral Frailty Group	Oral Frailty Group	*p*-Value
GNRI, %				0.962
No risk	31.1	31.5	30.6	
Low risk	25.9	27.4	24.2	
Moderate risk	31.9	30.1	33.9	
High risk	11.1	11.0	11.3	
NRI-JH, %				0.933
Low risk	86.7	87.7	85.5	
Medium risk	8.9	8.2	9.7	
High risk	4.4	4.1	4.8	
MNA-SF, %				0.296
Normal	56.3	60.3	51.6	
Risk of malnutrition	43.0	38.4	48.4	
Malnutrition	0.7	1.3	0	
Sarcopenia, %				0.059
Normal	39.2	42.5	35.5	
Sarcopenia	28.9	34.2	22.6	
Severe sarcopenia	31.9	23.3	41.9	
Revised J-CHS, %				0.0006
Normal	12.6	13.7	11.3	
Prefrailty	54.1	67.1	38.7	
Frailty	33.3	19.2	50.0	

GNRI, Geriatric Nutritional Risk Index; J-CHS, Japanese version of the Cardiovascular Health Study; MNA-SF, Short-Form Mini-Nutritional Assessment; NRI-JH, Nutritional Risk Index for Japanese Hemodialysis Patients.

**Table 5:** Patient characteristics at baseline in all patients aged <65 years and in the oral frailty and non-oral frailty groups.

Variable	All Patients	Non-Oral Frailty Group	Oral Frailty Group	*p*-Value
Patients, *n* (male %)	66 (80.3)	50 (76.0)	16 (93.8)	0.162
Age, years	54.3 ± 7.8	53.2 ± 8.1	57.9 ± 5.8	0.036
Duration of dialysis, months	82 (31–145)	80 (31–154)	84 (45–123)	0.899
Dialysis time, min	244 ± 21	244 ± 20	244 ± 24	0.955
Body mass index, kg/m^2^	24.0 ± 4.6	24.7 ± 4.4	22.0 ± 4.6	0.055
Systolic blood pressure, mmHg	164 ± 25	164 ± 27	166 ± 20	0.699
Diastolic blood pressure, mmHg	90 ± 13	90 ± 11	88 ± 16	0.446
Heart rate, bpm	78 ± 10	78 ± 9	77 ± 13	0.356
Serum urea nitrogen, mg/dL	60.4 ± 14.3	59.9 ± 12.2	62.0 ± 19.9	0.616
Creatinine, mg/dL	11.2 ± 2.8	11.6 ± 2.8	10.1 ± 2.7	0.068
Hemoglobin, g/dL	11.0 ± 1.2	11.0 ± 1.1	10.9 ± 1.3	0.966
Albumin, g/dL	3.7 ± 0.3	3.7 ± 0.2	3.6 ± 0.5	0.798
Corrected calcium, mg/dL	9.0 ± 0.7	9.1 ± 0.7	8.9 ± 0.6	0.408
Phosphate, mg/dL	3.7 ± 0.3	3.7 ± 0.2	3.6 ± 0.5	0.708
Intact PTH, pg/mL	148 (112–202)	142 (110–208)	150 (134–180)	0.852
C-reactive protein, mg/dL	0.11 (0.07–0.29)	0.14 (0.07–0.31)	0.09 (0.07–0.21)	0.410
Total iron binding capacity, μg/dL	290 ± 68	294 ± 67	276 ± 72	0.445
Total cholesterol, mg/dL	165 ± 38	169 ± 39	153 ± 33	0.164
spKt/V	1.43 ± 0.2	1.43 ± 0.2	1.43 ± 0.2	0.946
Cause of ESKD, *n* (%)				
Hypertension	18 (27.3)	15 (22.7)	3 (4.5)	0.463
Diabetic nephropathy	34 (51.5)	23 (34.8)	11 (16.7)	
Chronic glomerular nephritis	8 (12.1)	7 (10.6)	1 (1.5)	
Others	6 (9.1)	5 (7.6)	1 (1.5)	
Comorbidities, *n* (%)				
Diabetes mellitus	34 (51.5)	23 (46.0)	11 (68.8)	0.154
Ischemic heart disease	18 (27.3)	13 (26.0)	5 (31.3)	0.751
Cerebrovascular disease	21 (31.8)	16 (32.0)	5 (31.3)	0.998
Osteoporosis	10 (15.2)	6 (12.0)	4 (25.0)	0.242

ESKD, end-stage kidney disease; PTH, parathyroid hormone.

**Table 6:** Risk categories for nutritional status, sarcopenia, and physical frailty at baseline in all patients aged <65 years and in the oral frailty and non-oral frailty groups.

Indicator	All Patients	Non-Oral Frailty Group	Oral Frailty Group	*p*-Value
GNRI, %				0.021
No risk	62.1	70.0	37.5	
Low risk	18.2	16.0	25.0	
Moderate risk	16.7	14.0	25.0	
High risk	3.0	0	12.5	
NRI-JH, %				0.139
Low risk	92.5	96.0	81.3	
Medium risk	4.5	2.0	12.5	
High risk	3.0	2.0	6.2	
MNA-SF, %				0.089
Normal	87.9	92.0	75.0	
Risk of malnutrition	10.6	8.0	18.8	
Malnutrition	1.5	0	6.2	
Sarcopenia, %				0.002
Normal	81.8	90.0	56.3	
Sarcopenia	13.6	10.0	25.0	
Severe sarcopenia	4.6	0	18.7	
Revised J-CHS, %				0.002
Normal	45.5	54.0	18.8	
Prefrailty	47.0	44.0	56.2	
Frailty	7.5	2.0	25.0	

GNRI, Geriatric Nutritional Risk Index; J-CHS, Japanese version of the Cardiovascular Health Study; MNA-SF, Short-Form Mini-Nutritional Assessment; NRI-JH, Nutritional Risk Index for Japanese Hemodialysis Patients.

## Data Availability

The data used in this study are available from the corresponding author.

## Supplementary Materials

The following supporting information can be downloaded at https://www.mdpi.com/article/10.3390/nu16203467/s1, Figure S1: AWGS 2019 algorithm for sarcopenia; Table S1: Oral Frailty Index-8 categories; Table S2: Geriatric Nutritional Risk Index categories, Table S3: Nutritional Risk Index for Japanese Hemodialysis Patients categories; Table S4: Scoring on the Short-Form Mini-Nutritional Assessment; Table S5: Categories of sarcopenia defined by the Asian Working Group for Sarcopenia 2019; Table S6: Revised Japanese version of the Cardiovascular Health Study criteria. Table S7: Univariate and multivariate logistic regression analyses of risk factors for oral frailty.
