# Supplementary material for: Oral Frailty as a Risk Factor for Malnutrition and Sarcopenia in Patients on Hemodialysis: A Prospective Cohort Study
Source: Nutrients. 2024 Oct 13;16(20):3467. doi: 10.3390/nu16203467 (PMC11510359; doi:10.3390/nu16203467)
Supplement: Supplementary file 1 [file nutrients-16-03467-s001.zip › nutrients-3179668-supplementary.pdf]

Supplementary Table S1. Oral Frailty Index-8 categories

Question	Yes	No
Do you have any difficulties eating tough foods compared with 6 months ago?	2	0
Have you choked on tea or soup recently?	2	0
Do you wear a denture?	2	0
Do you often experience dry mouth?	1	0
Do you go out less frequently than you did 6 months ago?	1	0
Can you eat foods as hard as squid jerky or pickled radish?	0	1
Do you brush your teeth at least twice a day?	0	1
Do you visit a dental clinic at least once a year?	0	1

Total Score	Risk Category
0–2	Low
3	Moderate
4–11	High

Supplementary Table S2. Geriatric Nutritional Risk Index categories

**Total Score**	**Risk Category**
≥98	None
≥92, <98	Low
≥82, <92	Moderate
<82	High

Supplementary Table S3. Nutritional Risk Index for Japanese hemodialysis patient categories

Low body mass index* (<20 kg/m^2)	Yes = 3		No = 0
Low serum albumin level<br>-Under 65 years (<3.4 g/dL)<br>-65 years or older (<3.2 g/dL)	Yes = 4		No = 0
Serum total cholesterol level	<130 mg/dL = 1	≥130 mg/dL,<br><220 mg/dL = 0	≥220 mg/dL = 2
Low serum creatinine level<br>-Female, under 65 years (<9.7 mg/dL)<br>-Female, 65 years or older (<8.0 mg/dL)<br>-Male, under 65 years (<11.6 mg/dL)<br>-Male, 65 years or older (<9.7 mg/dL)	Yes = 4		No = 0

**Total Score**	**Risk Category**
0–7	Low
8–10	Medium
11–13	High

Supplementary Table S4. Scoring on the Short-Form Mini-Nutritional Assessment

How much has your food intake decreased in the past 3 months because of loss of appetite, digestive problems, or chewing or swallowing difficulties?	Severe loss of appetite = 0	Moderate loss of appetite = 1	No loss of appetite = 2	
How much weight have you lost in the past 3 months?	≥3 kg = 0	Do not know = 1	1–3 kg = 2	No weight loss = 3
How far can you walk by yourself?	Bedridden or chairbound = 0	Capable of getting out of bed or a chair but cannot go out = 1	Capable of going out = 2	
Have you experienced psychological stress or an acute illness in the past 3 months?	Yes = 0		No = 2	
Neuropsychiatric problems	Severe dementia or depression = 0	Mild dementia = 1	No such problems = 2	
Body mass index*, kg/m^2	<19 = 0	≥19, <21 = 1	≥21, <23 = 2	≥23 = 3

**Total Score**	**Evaluation Stage**
12–14	Normal, no need for further assessment
8–11	Risk of malnutrition
0–7	Malnutrition

Supplementary Table S5. Categories of sarcopenia defined by the Asian Working Group for Sarcopenia 2019

Low muscle mass + low muscle strength + low physical performance	Severe sarcopenia
Low muscle mass + low muscle strength/low physical performance	Sarcopenia
Except for above	Normal

Supplementary Table S6. Revised Japanese version of the Cardiovascular Health Study criteria

Question	Yes	No
Weight loss: Unintentional weight loss of ≥2 kg in the past 6 months	1	0
Muscle weakness: handgrip, <28.0 kg for men and <18.0 kg for women	1	0
Exhaustion: feeling tired for no reason for the past 2 weeks	1	0
Slowness: Walking <1 m/s	1	0
Low activity:<br>① Do you do light exercise/gymnastics<br>② Do you do regular exercise/sports?<br>When patients are asked questions ① and ②,<br>their answer is "I don't do it once a week".	1	0

**Total Score**	**Category**
0 point	Normal
1–2 points	Prefrailty
3–5 points	Frailty

Supplementary Table S7. Univariate and multivariate logistic regression analyses of risk factors for oral frailty.

Variables	Univariate					Multivariate (R^2 = 0.69)				
	Estimate	SE	95%CI		P value	Estimate	SE	95%CI		P value
			Lower	Upper				Lower	Upper	
Female sex	-0.078	0.173	-0.419	0.263	0.652					
Age	0.078	0.011	0.057	0.100	< 0.0001	0.056	0.015	0.026	0.086	0.0003
Duration of dialysis	-0.003	0.002	-0.007	0.001	0.187					
Diabetes mellitus	0.267	0.158	-0.040	0.580	0.092	0.164	0.151	-0.134	0.462	0.279
Ischemic heart disease	0.077	0.168	-0.254	0.408	0.646					
Cerebrovascular disease	0.164	0.161	-0.153	0.482	0.310					
Osteoporosis	0.495	0.156	0.187	0.803	0.002	-0.075	0.164	-0.399	0.247	0.645
Body mass index	-0.062	0.038	-0.138	0.012	0.101					
Systolic blood pressure	0.010	0.006	-0.002	0.023	0.106					
Heart rate	-0.011	0.013	-0.038	0.016	0.425					
Serum urea nitrogen	-0.017	0.011	-0.039	0.003	0.099	-0.007	0.010	-0.026	0.012	0.483
Creatinine	-0.204	0.053	-0.310	-0.098	0.0002	0.098	0.083	-0.064	0.262	0.235
Hemoglobin	-0.027	0.133	-0.291	0.236	0.839					
Albumin	-1.445	0.407	-2.248	-0.643	0.001	-0.465	0.556	-1.563	0.632	0.404
Corrected calcium	0.029	0.234	-0.431	0.491	0.899					
Phosphate	-0.007	0.122	-0.248	0.232	0.951					
Intact PTH	-0.003	0.002	-0.007	0.001	0.057	-0.002	0.001	-0.005	0.001	0.259
C-reactive protein	0.082	0.117	-0.149	0.313	0.485					
TIBC	-0.002	0.003	-0.008	0.002	0.291					
Total cholesterol	-0.001	0.005	-0.021	-0.003	0.009	-0.006	0.004	-0.014	0.002	0.177

spKt/V	-0.715	0.661	-2.017	0.586	0.279					
GNRI	-0.048	0.015	-0.078	-0.019	0.0014	0.029	0.028	-0.027	0.086	0.303
NRI-JH	0.153	0.050	0.054	0.252	0.0025	0.033	0.076	-0.116	0.183	0.660
MNA-SF	-0.346	0.084	-0.511	-0.181	< 0.0001	-0.144	0.122	-0.385	0.096	0.239
Sarcopenia	1.155	0.174	0.811	1.499	< 0.0001	0.276	0.264	-0.245	0.798	0.296
Revised J-CHS	0.852	0.115	0.624	1.081	< 0.0001	0.405	0.177	0.005	0.756	0.023

GNRI, Geriatric Nutritional Risk Index; J-CHS, Japanese version of the Cardiovascular Health Study; MNA-SF, Short-Form Mini-Nutritional Assessment; NRI-JH, Nutritional Risk Index for Japanese Hemodialysis Patients; PTH, parathyroid hormone; TIBC, total iron binding capacity.

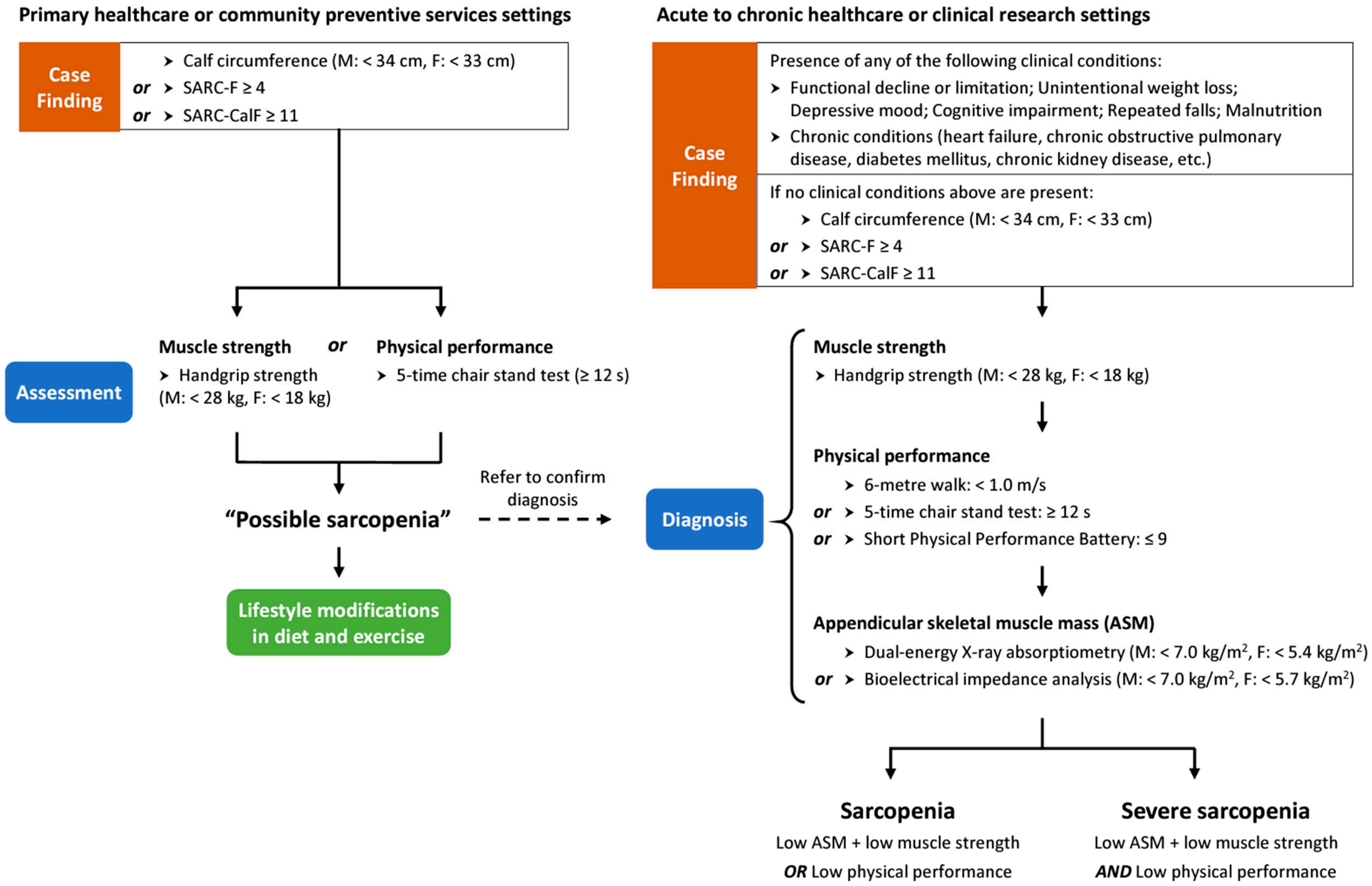

Supplementary Figure S1. AWGS 2019 algorithm for sarcopenia
